# Thermal Performance Improvement of AlGaN/GaN HEMTs Using Nanocrystalline Diamond Capping Layers

**DOI:** 10.3390/mi13091486

**Published:** 2022-09-07

**Authors:** Huaixin Guo, Yizhuang Li, Xinxin Yu, Jianjun Zhou, Yuechan Kong

**Affiliations:** 1 Science and Technology on Monolithic Integrated Circuits and Modules Laboratory, Nanjing 210016, China; 2 Nanjing Electronic Devices Institute, Nanjing 210016, China

**Keywords:** AlGaN/GaN HEMTs, nanocrystalline diamond capping layers, thermal resistance, output characteristic

## Abstract

Nanocrystalline diamond capping layers have been demonstrated to improve thermal management for AlGaN/GaN HEMTs. To improve the RF devices, the application of the technology, the technological approaches and device characteristics of AlGaN/GaN HEMTs with gate length less than 0.5 μm using nanocrystalline diamond capping layers have been studied systematically. The approach of diamond-before-gate has been adopted to resolve the growth of nanocrystalline diamond capping layers and compatibility with the Schottky gate of GaN HEMTs, and the processes of diamond multi-step etching technique and AlGaN barrier protection are presented to improve the technological challenge of gate metal. The GaN HEMTs with nanocrystalline diamond passivated structure have been successfully prepared; the heat dissipation capability and electrical characteristics have been evaluated. The results show the that thermal resistance of GaN HEMTs with nanocrystalline diamond passivated structure is lower than conventional SiN-GaN HEMTs by 21.4%, and the mechanism of heat transfer for NDC-GaN HEMTs is revealed by simulation method in theory. Meanwhile, the GaN HEMTs with nanocrystalline diamond passivated structure has excellent output, small signal gain and cut-off frequency characteristics, especially the current–voltage, which has a 27.9% improvement than conventional SiN-GaN HEMTs. The nanocrystalline diamond capping layers for GaN HEMTs has significant performance advantages over the conventional SiN passivated structure.

## 1. Introduction

AlGaN/GaN high-electron mobility transistors (GaN HEMTs) play a central role for RF power electronics applications. However, they are influenced by channel temperature, which is caused by increased energy transfer from electrons to the lattice at high power; as the power density in GaN HEMTs rises, the heat removal near the junction regions of GaN HEMTs becomes increasingly important for device performance and reliability [1,2,3,4,5,6,7,8,9,10]. To reduce channel temperature, several approaches have been proposed for thermal management, The obvious solution is to use high thermal conductive material immediately adjacent to the hot spot of the chip to minimize temperature rise [5,6,7,8,9,10,11,12,13,14]. Heat-spreading schemes have involved growth of AlGaN/GaN on single crystal or CVD diamond substrates or capping of fully processed HEMTs surface using nanocrystalline diamond capping layers [1,4,7,15,16,17]. However, the approaches with bottom-side diamond substrates have faced limitations with substrate size and the bonding layer of nitride material. Further, the method of introducing a heat-spreading films into the top-side structure has been proposed more recently; one such material, nanocrystalline diamond, possesses a high thermal conductivity and is expected to be a suitable candidate for a heat-spreading material [10,11,12,13,14,15,16,17,18]. The dissipation structure of nanocrystalline diamond capping layers (NDC) for the GaN HEMTs has provided relevant research results. U.S. Naval Research Laboratory developed the NDC-GaN devices that had a 20% lower channel temperature at equivalent power dissipation, but it is difficult to be applied to RF devices because the gate finger length is greater than 2 μm [1,6]. Fujitsu Laboratories Ltd. has deposited diamond films on the front surface of the HEMT device and made it a priority development project with a view toward a reduction in generated heat of 40%. Previous simulation and experiment work in the above research has largely concentrated in the fabrication of NDC and analysis of temperature and electrical performance [2,3,4,5,6,7,8,9]. However, a systematic process study of NDC-GaN HEMTs for device applications has not been thoroughly previously investigated thus far, this will continue to face challenges from the growth of NDC, diamond etching in the gate area, the compatibility of gate preparation and all the rest.

In this paper, the thermal management AlGaN/GaN HEMTs using NDC layers has been studied systematically. The approach of diamond-before-gate was adopted, the processes of diamond etching and AlGaN barrier protection were developed, and the first NDC-GaN HEMTs with gate length less than the 0.5 μm was successfully prepared. Meanwhile, characteristics, such as heat dissipation capability, current–voltage, transfer curves, small signal gain and cut-off frequency, were tested and evaluated.

## 2. Experimental Details

**Figure 1:**
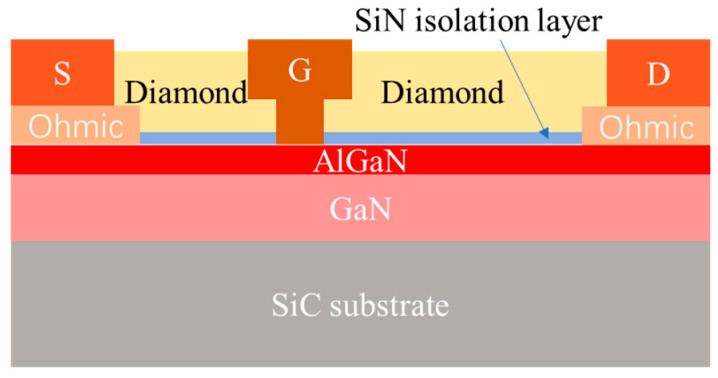
Schematic of the GaN HEMTs with the nanocrystalline diamond capping layers (NDC).

Here, the approach of diamond-before-gate was adopted to resolve the growth of NDC layers and compatibility with the Schottky gate of GaN devices, and the processes of diamond etching and AlGaN barrier protection were developed to improve the technological challenge of gate metal length less than the 0.5 μm for RF devices applications. Based on a traditional SiC substrate GaN HEMTs device, the NDC-GaN HEMTs was designed as double gate fingers structure, the length and width of gate were, respectively, 0.3 μm and 125 μm, the total gate width was 250 μm. In addition, the NDC thickness was designed as 500 nm with a 20 nm SiN isolation layer for an efficient heat dissipation, as illustrated in Figure 1.

**Figure 2:**
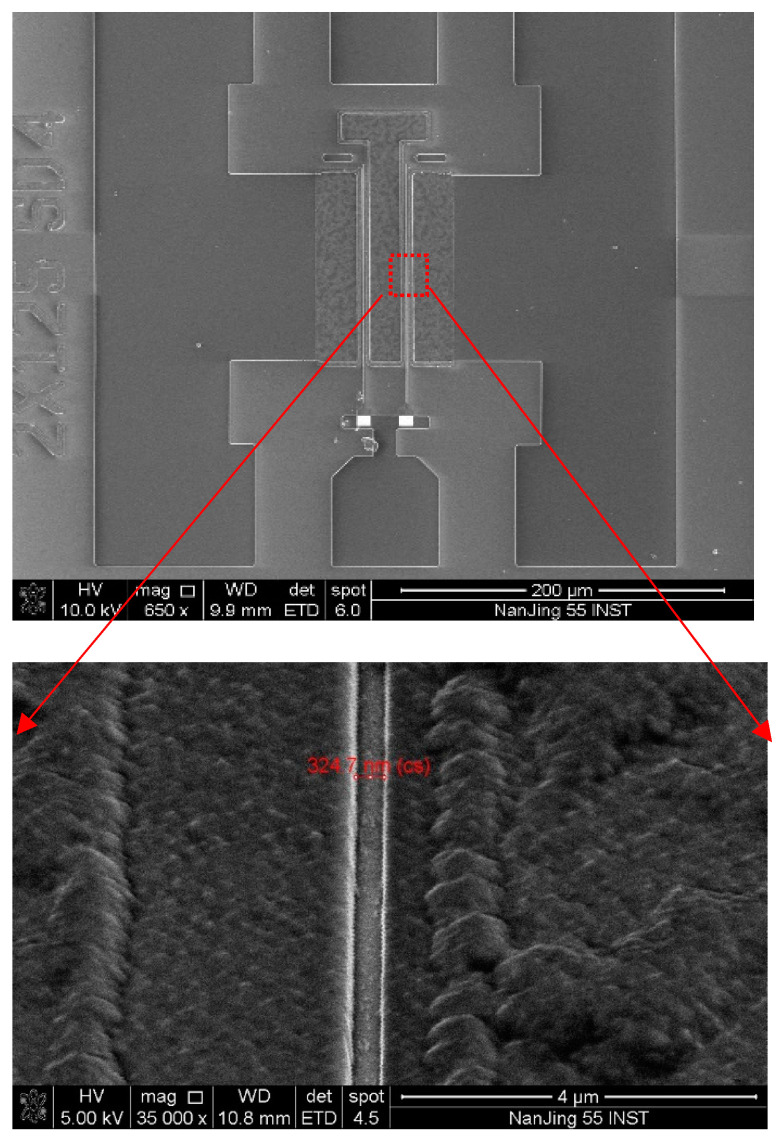
Etching quality of the NDC for gate region with 0.3 µm.

The specific process of preparing NDC-GaN HEMTs was as follows. The device was carried out on a small piece with a size of 1 cm^2^. First, mesa isolation and ohmic metal were performed by conventional operations; the thickness of source and drain was 200 nm. Then, the isolation layer of SiN was adopted, with the purpose of protecting AlGaN barrier from being broken; while in the processes of diamond growth and diamond etching, the thickness of isolation layer was 20 nm and was deposited by plasma enhanced chemical vapor deposition, which had a function of not only achieving protection but also avoiding large interfacial thermal resistance. Second, the nanocrystalline diamond capping layers (NDC) was grown at 710 °C by microwave chemical vapor deposition (CVD) method; this growth temperature effectively guaranteed ohmic metal stability of source and drain. The growth rate of NDC was controlled in 80 nm/h, the lower growth rate was for the high quality growth of NDC and ensured a high thermal conductivity of NDC; the NDC thickness was 500 nm. Third, diamond etching technology was studied for gate region. A 150 nm SiN film was accepted as a hard mask for the diamond etch process; the SiN film was deposited by PECVD method. The etching was extremely difficult because of gate length less than the 0.5 μm, so the multi-step etching technique was adopted for the NDC of gate region. The first step was to employ rapid etching by ICP with O_2_/Ar atmospheres, the verticality of etching was guaranteed, almost 80 percent of NDC was etched. The second step was for high quality etching of the surface using ICP with O_2_ atmosphere; this step can ease the burrs of etching surface. The third step was etching the isolation layer, in which low power, O_2_ atmosphere and over etching were adopted; the etching conditions of ICP can not only guarantee SiN etching completely but also avoid etching damage of AlGaN barrier. The high quality gate region had been obtained by the multi-step etching technique, shown in Figure 2, using the scanning electron microscopy (SEM) method. Fourth, Schottky gate was prepared by an e-beam-evaporated method; the actual gate length was 0.3247 μm, and gate thickness was 520 nm. Finally, the metal interconnection for source, gate, drain and circuit was implemented with Au through deposition method. The NDC-GaN HEMTs with 0.3 μm gate width was successfully developed, in order to compare NDC-GaN HEMTs with traditional structure GaN HEMTs and the GaN HEMTs with SiN passivated structure was prepared.

**Figure 3:**
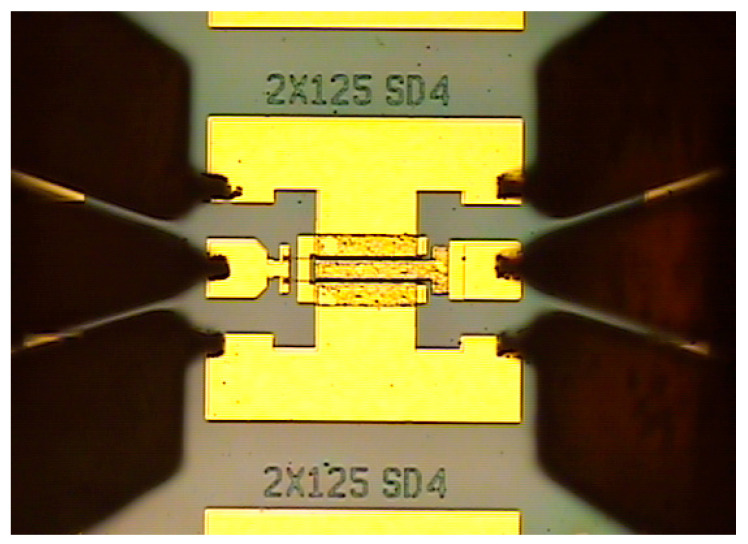
Optical images of NDC-GaN HEMTs when electrical properties are tested.

The aims at resolving heat accumulation effect and improving electrical properties of GaN HEMTs through the method of capping of fully processed GaN HEMTs surface using the NDC, and the electrical properties, such as current-voltage, gate leakage, transfer curves, small signal gain and cut-off frequency, were performed with a B1500A Semiconductor Device Analyzer, as shown in Figure 3. Meanwhile, the heat dissipation capability was evaluated using an IR thermal photogrammetry; the model is QFI Infrascope TM-HST, the spatial resolution of the model is 2.7 μm and is the highest by far. These electrical output properties and heat dissipation characteristics of the NDC-GaN HEMTs were compared with those of GaN HEMTs with SiN passivated structures, admittedly, the purpose was to show that the NDC has an excellent property for the AlGaN/GaN HEMTs.

## 3. Results and Discussion

### 3.1. Analysis of Output and Transfer Characteristics

**Figure 4:**
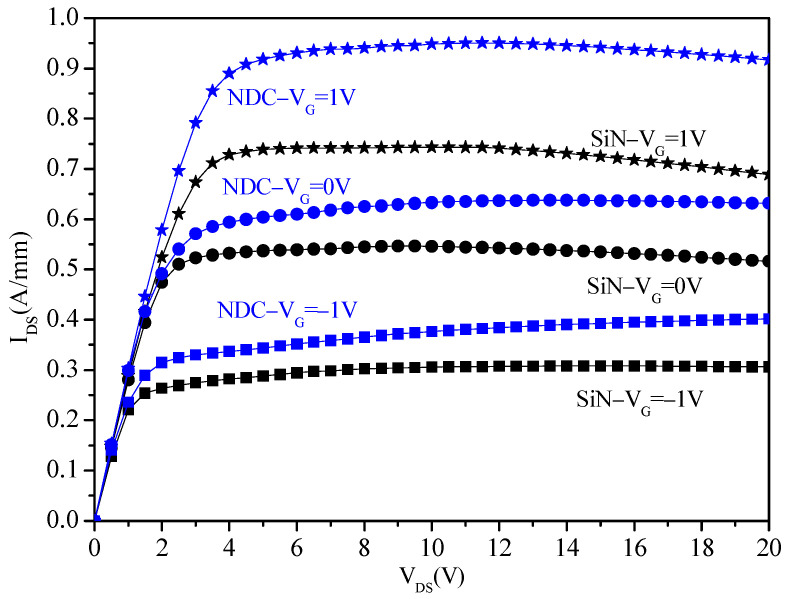
Output characteristic of GaN HEMTs with NDC and SiN passivated structures.

The output and transfer characteristics are primary indicators to measure the quality of GaN HEMTs, so the current–voltage (I_DS_-V_DS_) and transfer curves (I_DS_-V_GS_) of GaN HEMTs with NDC and SiN passivated structures were tested and analyzed. The drain current is measured over V_DS_, which was from 0 V to 20 V at the fixed V_GS_ of −1 V, 0 V and 1 V. The result of output characteristic is shown in Figure 4. The self-heating phenomena occurs in the saturation region of output characteristic and is improved significantly in the GaN HEMTs with NDC passivated structure, as the V_GS_ increases, the improvement increases. At V_GS_ = 1 V, the maximum drain current (I_DS_) of the NDC-GaN HEMTs is 950.45 mA/mm, and the maximum I_DS_ of conventional SiN-GaN HEMTs is 743.28 mA/mm; the result indicates a 27.9% improvement in output characteristic. The improvement of drain current of the NDC-GaN HEMTs is due to the less degradation of temperature dependent mobility and suggests either that traps at the GaN HEMTs surface are better passivated by using a 20 nm SiN and 500 nm NDC passivated structure than by using a 200 nm thick SiN passivated structure.

**Figure 5:**
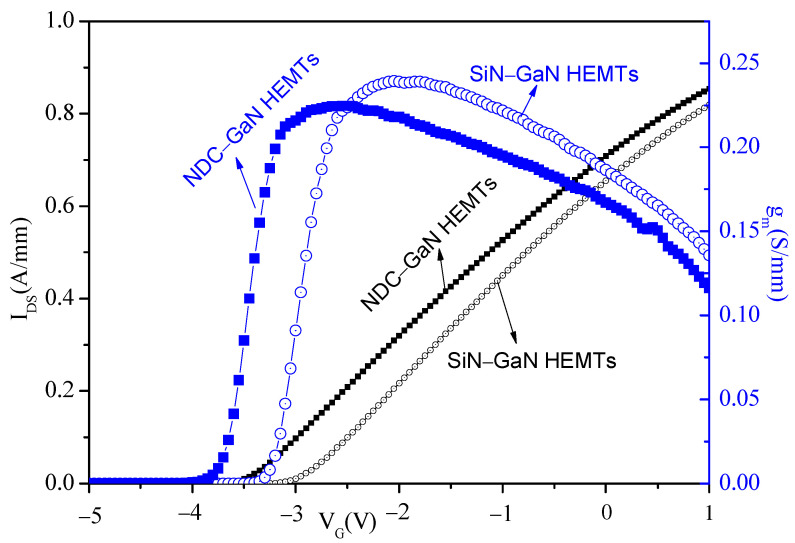
Transfer characteristic of GaN HEMTs with NDC and SiN passivated structure; the black curves represent transfer curves, and the green curves represent transconductance.

Furthermore, the results of transfer curves (I_DS_-V_GS_) characteristic of GaN HEMTs with NDC and SiN passivated structures are shown in Figure 5 (black curves); the I_DS_-V_GS_ was measured over gate voltage, which was from −5 V to 1 V. The threshold voltages (V_T_) of GaN HEMTs with NDC and SiN passivated structures are, respectively, 3.45 V and 3 V; the slight negative V_T_ shift can be attributed to the higher 2DEG density from the increased tensile strain in the heterostructure, as well as reduced self-heating. In addition, Transconductance (g_m_) of GaN HEMTs has an impact on device linearity and noise, which are important parameters in RF devices. Therefore, the g_m_ of GaN HEMTs with NDC and SiN passivated structures are analyzed and shown in Figure 5 (green curves). From Figure 5, the peak values of g_m_ for GaN HEMTs with NDC and conventional SiN passivated structure are 0.225 S/mm and 0.239 S/mm, respectively. The transconductance of NDC-GaN HEMTs is lower than conventional SiN-GaN HEMTs by 5.86%. It is due to the increased tensile strain effect in the NDC-GaN HEMTs because of the growth of NDC layers.

### 3.2. Analysis of Small Signal Gain and Cut-Off Frequency Characteristics

**Figure 6:**
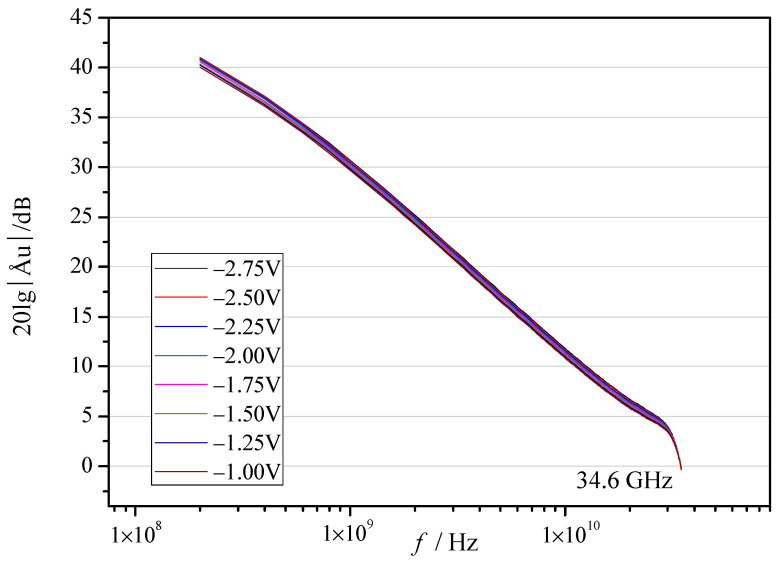
Small signal gain and cut-off frequency characteristics of NDC-GaN HEMTs.

**Figure 7:**
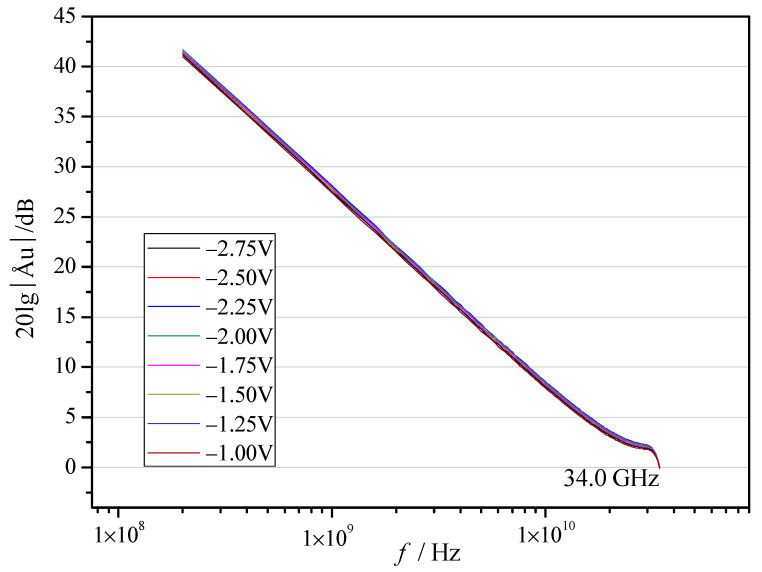
Small signal gain and cut-off frequency characteristics of conventional SiN-GaN HEMTs.

The primary advantages of GaN devices are higher power density and higher cut-off frequency; the increase in power density for NDC-GaN HEMTs has been reported by test results of output characteristic, so cut-off frequency of GaN HEMTs with NDC and SiN passivated structures were measured and analyzed; the gate voltage was from −2.75 V to −1 V with a step of 0.25 V; the results are shown in Figure 6 and Figure 7 and Table 1. Figure 6 shows the cut-off frequency of NDC-GaN HEMTs is 34.6 GHz; Figure 7 shows the cut-off frequency of conventional SiN-GaN HEMTs is 34.0 GHz; the cut-off frequency of NDC-GaN HEMTs is higher than conventional SiN-GaN HEMTs by 1.8% (Table 1). Moreover, the most notable improvement is small signal gain, the trend of small signal gain curves of GaN HEMTs with NDC and SiN passivated structures are almost the same; at the same frequency, the small signal gain of NDC-GaN HEMTs is higher than that of conventional SiN-GaN HEMTs. When the frequency is 10 GHz, the small signal gain of GaN HEMTs with NDC and SiN passivated structure are between 10.83 dB and 11.80 dB and between 7.91 dB and 8.55 dB, respectively, the average gain of NDC-GaN HEMTs is higher than conventional SiN-GaN HEMTs by 36.7% (Table 1). For the GaN RF devices, the higher the cut-off frequency, the bigger the small signal gain, or at least better performance.

### 3.3. Analysis of Heat Dissipation Capability

**Figure 8:**
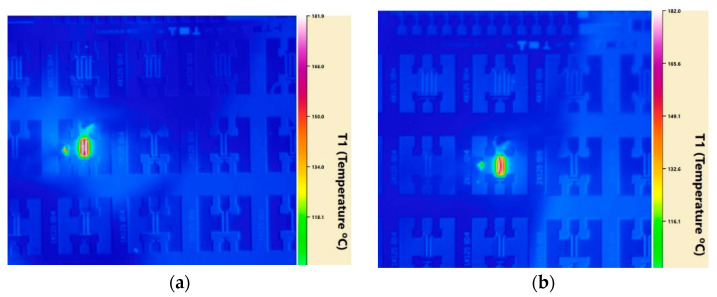
Junction temperature of GaN HEMTs with NDC (**a**) and SiN (**b**) passivated structure measured by IR thermal photogrammetry.

**Figure 9:**
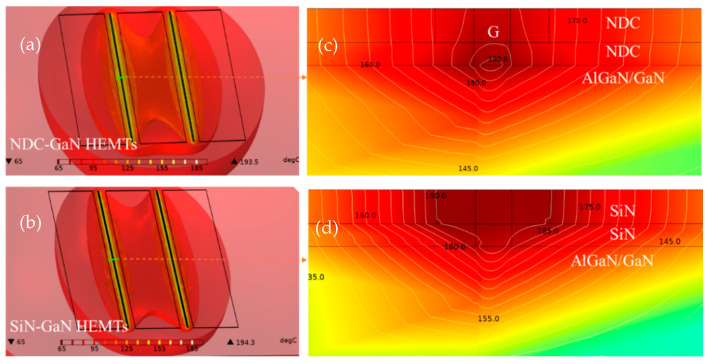
The temperature distribution and junction temperature of GaN HEMTs with NDC (**a**) and SiN (**b**) passivated structures of thermal simulation calculations; the temperature gradient around heat source regions (the center regions of gate) of GaN HEMTs with NDC (**c**) and SiN (**d**) passivated structures.

In theory, the nanocrystalline diamond capping layers provides a high thermal conductivity path for GaN devices and reduces the junction temperature of the GaN HEMTs; the estimation of heat dissipation capability is important to NDC-GaN HEMTs applications. Therefore, we have the IR thermal photogrammetry; the emissivity coefficients need to be recalibrated for each thermal test, therefore, it is not affected by surface capped materials of GaN HEMEs. In addition, we used the 5× infrared objective for high spatial resolution, in order to measure the device thermal resistance (*R)* of GaN HEMTs with NDC and SiN passivated structure. For comparability, the power (*P_diss_*) was tested under the same junction temperature (*T_j_*) and ambient temperature (*T_a_* = 65 °C), and the junction temperature (*T_j_*) was controlled in 180 °C to 185 °C by adjusting the power (*P_diss_*) of GaN device; the results are shown in Figure 8. The power (*P_diss_*) of GaN HEMTs with NDC and SiN passivated structures are, respectively, 5.69 W and 4.48 W, corresponding to the junction temperature (*T_j_*) of 181.9 °C and 182 °C (Figure 9). According to the calculating formula as *R* = *(T_j_* − *T_a_*)/*P_diss_*, the device thermal resistance (*R*) of GaN HEMTs with NDC and SiN passivated structures are 20.54 K/W and 26.12 K/W, respectively. The lower proportion of *R* is 21.4%, which is due to the effective spreading and conduction of the heat from the hot-spot by the nanocrystalline diamond capping layers; the enhanced heat spreading feature of NDC-GaN HEMTs makes it more reliable and durable than conventional GaN HEMTs. The results suggest that the NDC has a significant impact on the heat transfer paths for GaN HEMTs application.

To understand of the mechanism of NDC to thermal management of AlGaN/GaN HEMTs, the temperature distribution of GaN HEMTs with NDC and SiN passivated structures is analyzed using the finite element method [18]. The simulation models were designed to accord with NDC and SiN passivated GaN HEMTs, and simulated conditions were based on actual test conditions. The results are shown in Figure 9, Figure 9a,b show the temperature distribution and junction temperature of GaN HEMTs. The simulate values of junction temperature are below the experimental values (Figure 8); the deviation is less than 10%, this is primarily due to the spatial resolution and surface temperature of IR thermal photogrammetry; and the correctness and validation of the simulation model is demonstrated. Meanwhile, the temperature distribution of SiN-GaN HEMTs is more focused than that of the NDC-GaN HEMTs; it suggests that the NDC effective solves heat accumulation in GaN HEMTs. To identify the cause of heat dissipating capacity with NDC passivated structure, the temperature gradient (yellow lines) around heat source regions (the center regions of gate) is analyzed in Figure 9c,d. the temperature gradient of NDC-GaN HEMTs is less than that of NDC-GaN HEMTs, especially the NDC layer; this indicates that the NDC plays a key functional role for thermal spreader. The simulation results demonstrate the path and mechanism of heat transfer for NDC-GaN HEMTs in theory and are consistent with experiments results.

## 4. Conclusions

In this paper, device technologies and performances of the AlGaN/GaN HEMTs using NDC layers have been studied systematically. The approach of diamond-before-gate is adopted to resolve the growth of NDC layers and compatibility with the Schottky gate of GaN devices; three-step diamond etching techniques and 20 nm SiN isolation layer are developed to improve the technological challenge of the gate length less than the 0.5 μm and AlGaN barrier protection. The 0.3 μm gate length GaN HEMTs with NDC and conventional SiN passivated structures have been successfully prepared, meanwhile, the heat dissipation capability and electrical characteristics have been evaluated. Thermal test result shows that thermal resistance of NDC-GaN HEMTs is lower than conventional SiN-GaN HEMTs by 21.4% and suggests that the NDC has a significant impact on the heat transfer paths for GaN HEMTs application; the mechanism of the heat transfer for NDC-GaN HEMTs is revealed by simulation method and is consistent with experiment result. Not only that, the NDC-GaN HEMTs has better output, smaller signal gain and cut-off frequency characteristics than those of SiN-GaN HEMTs; the cut-off frequency is 34.6 GHz, which is higher than SiN-GaN HEMTs by 1.8%, especially the current–voltage (V_GS_ = 1 V) and small signal gain (10 GHz), which show, respectively, a 27.9% and 36.7% improvement. Overall, this study shows that the nanocrystalline diamond capping layers have excellent properties for AlGaN/GaN HEMTs in RF application.

## Figures and Tables

**Table 1:** Small signal gain and cut-off frequency characteristics.

Definition	Small Signal Gain (10 GHz)	Cut-Off Frequency
NDC-GaN HEMTs	10.83–11.80 dB	34.6 GHz
SiN-GaN HEMTs	7.91–8.55 dB	34.0 GHz

## Data Availability

Not applicable.
